# A multimodal machine-learning diagnostic model for Alzheimer’s disease: integrating OCTA-derived retinal microvascular features and clinical variables

**DOI:** 10.3389/fnagi.2026.1911592

**Published:** 2026-09-23

**Authors:** Qifeng Zhou, Zhongping Tian, Wei Zhao, Xi Chen, Yuan Liu, Tengxiao Wang, Jiaxuan Chen, Yanlong Bi, Li Zhang

**Affiliations:** 1 Department of Ophthalmology, Tongji Hospital, School of Medicine, Tongji University, Shanghai, China; 2 School of Public Health, Zunyi Medical University, Zunyi, China; 3 Department of Neurology, Shanghai Putuo People's Hospital, Tongji University, Shanghai, China; 4 Clinical Research Center, Tongji Hospital, School of Medicine, Tongji University, Shanghai, China; 5 School of Medicine, Tongji University, Shanghai, China

**Keywords:** Alzheimer’s disease, machine learning, optical coherence tomography angiography, retinal biomarkers, XGBoost

## Abstract

**Introduction:**

Alzheimer’s disease (AD) lacks widely accessible, noninvasive approaches for the evaluation of AD remain limited. We developed a multimodal machine-learning model that integrates retinal microvascular features derived from optical coherence tomography angiography (OCTA) with clinical variables to classify AD.

**Methods:**

In this cross-sectional case–control study, 160 participants (81 healthy controls and 79 patients with AD) underwent macular OCTA imaging. Twenty-seven superficial capillary plexus features, including vessel density (VD), perfusion density (PD), and foveal avascular zone (FAZ) metrics, were analyzed. Logistic regression, support vector machine, random forest, and XGBoost classifiers were evaluated using stratified five-fold cross-validation. Feature selection was performed with nested cross-validated recursive feature elimination, and model interpretability was assessed with Shapley additive explanations (SHAP).

**Results:**

Patients with AD showed reduced VD and PD across multiple macular subfields. The multimodal random forest model achieved the highest area under the receiver operating characteristic curve (AUC = 0.853), whereas the multimodal XGBoost model achieved the highest sensitivity (0.800) with an AUC of 0.839. SHAP analysis identified whole-macula VD, nasal inner-ring VD, FAZ circularity, and inferior inner-ring PD as influential predictors.

**Conclusion:**

These findings provide proof-of-concept evidence that OCTA-derived retinal microvascular features, particularly when combined with selected clinical variables, may help distinguish clinically diagnosed AD from HC in this selected case–control cohort. Their utility for population screening or clinical diagnosis remains unestablished and requires prospective external validation in consecutive, clinically representative cohorts including mild cognitive impairment (MCI), other dementias, and relevant neurological and ocular differential diagnoses.

## 1 Introduction

AD is a neurodegenerative disorder and the most common cause of dementia. Its prevalence continues to rise, particularly in China, imposing substantial medical and socioeconomic burdens. As of 2021, approximately 17 million individuals in China were living with AD and other dementias, accounting for 29.8% of the global total. Associated treatment costs are projected to reach USD 1.8 trillion by 2050 (Ren et al., 2022).

However, early diagnosis of AD remains a major global challenge. AD is characterized by an insidious onset, with neuropathological changes occurring up to 20 years or more before the emergence of clinical symptoms. In addition, confirmatory tools such as positron emission tomography/computed tomography (PET-CT) and invasive cerebrospinal fluid (CSF) testing are difficult to implement widely in routine clinical practice. Therefore, developing affordable and noninvasive approaches for early identification of AD is an urgent priority.

The retina is considered an extension of the central nervous system (Jiang et al., 2018) and a window to the brain. OCTA enables precise, noninvasive quantification of retinal microcirculation, and multiple studies have reported reduced retinal microvascular perfusion in patients with AD compared with cognitively normal controls. Biscetti et al. (2021) showed that OCTA metrics, including vessel density (VD) in the superficial capillary plexus (SCP), intermediate capillary plexus (ICP), and deep capillary plexus (DCP), as well as perfusion density (PD) in the ICP, were significantly reduced in patients with mild cognitive impairment (MCI) and AD. Similarly, Chua et al. (2020) reported significantly decreased VD in both the SCP and DCP in patients with AD and mild cognitive impairment (MCI).

However, most ophthalmic artificial intelligence (AI) studies have relied on single-modality data. It remains unclear whether feature selection improves model generalizability and whether demographic and clinical variables add incremental diagnostic value beyond OCTA-derived features.

Accordingly, we developed and compared three machine-learning strategies: a baseline model using all OCTA-derived variables, an optimized model using selected retinal microvascular features, and a multimodal model combining selected OCTA features with demographic and clinical variables. We benchmarked four widely used machine-learning algorithms across these strategies to identify an efficient, noninvasive approach for AD detection.

## 2 Materials and methods

### 2.1 Participants

A total of 160 participants, comprising 79 patients with AD and 81 HC, were included between 2020 and 2023. Patients in the AD group presented to the Department of Neurology, Tongji Hospital, Tongji University, with self- or informant-reported cognitive decline. HC participants were patients who presented to the neurology or ophthalmology outpatient clinics for clinical care and were subsequently confirmed to be cognitively normal. Written informed consent was obtained from all participants before study enrollment.

The general inclusion criteria were as follows: (1) age between 50 and 90 years; (2) fulfillment of the group-specific diagnostic criteria described below; and (3) availability of macular optical coherence tomography angiography scans with a signal strength index >4.

Exclusion criteria were: (1) uncontrolled diabetes mellitus or hypertension, or ocular fundus diseases that could affect OCTA measurements, such as diabetic retinopathy; (2) high refractive error, defined as an absolute spherical equivalent ≥6 D; (3) intraocular surgery within the preceding 6 months; (4) a history of glaucoma or an intraocular pressure >21 mmHg; (5) macular or retinal disorders, including age-related macular degeneration, epiretinal membrane, or retinal vascular occlusion; (6) neurological disorders other than AD or severe psychiatric diseases; and (7) significant media opacity. Medical and neurological exclusion criteria were determined from the clinical history, medical records, neurological examination, laboratory investigations, and available neuroimaging findings. Ocular exclusion criteria were assessed using the ophthalmic history, refractive examination, intraocular pressure measurement, slit-lamp biomicroscopy, fundus examination, and OCTA images.

### 2.2 Neurological assessment and diagnostic classification

All participants underwent a comprehensive neurological examination and neuropsychological assessment. Global cognitive function was initially evaluated using the Montreal Cognitive Assessment-Basic (MoCA-B) and Mini-Mental State Examination (MMSE). Education-adjusted abnormality thresholds were applied. The MMSE thresholds were ≤17 for illiterate participants, ≤20 for participants with primary-school education, and ≤24 for those with secondary-school education or above. The corresponding MoCA-B thresholds were ≤19 for participants with primary-school education or less, ≤22 for those with secondary-school education, and ≤24 for those with university education. Scores at or below the corresponding thresholds were considered indicative of objective cognitive impairment.

Participants with objective cognitive impairment subsequently underwent functional assessment using the Activities of Daily Living Scale (ADL), in which higher scores indicate greater functional impairment. A total score ≥16 was used as a screening threshold for clinically relevant impairment in daily functioning. Because functional limitations may also result from noncognitive conditions, the ADL score was not used as a standalone diagnostic criterion for dementia. Dementia classification additionally required evidence that cognitive decline interfered with independent daily functioning, based on the clinical history, neurological assessment, and physician judgment.

Participants without dementia-level functional impairment, together with HC candidates, underwent domain-specific neuropsychological assessment covering memory, language, executive function, and visuospatial function. The assessment battery included the Hopkins Verbal Learning Test (HVLT), Wechsler Memory Scale (WMS), Boston Naming Test (BNT), Verbal Fluency Test (VFT), Shape Trail Test (STT), and Rey–Osterrieth Complex Figure Test (ROCFT). MCI was identified according to the quantitative neuropsychological criteria proposed by Bondi et al. (2014): impairment of at least two measures within the same cognitive domain, or impairment of at least one measure in each of two or more cognitive domains. Impairment was defined as performance more than 1 standard deviation below the relevant normative value. Participants meeting the criteria for MCI were excluded from the present AD-versus-HC analysis.

Classification into the AD group required objective cognitive impairment, cognitive-related loss of independence in daily functioning, and fulfillment of the 2011 National Institute on Aging–Alzheimer’s Association clinical criteria for probable AD dementia (McKhann et al., 2011). Diagnostic brain MRI was performed in 160 of 160 participants, whereas CSF AD biomarker testing was performed in 27 of 160 participants, including 25 of 79 patients with AD and 2 of 81 HC participants. CSF AD biomarkers and/or amyloid PET findings were considered when available but were not mandatory because these examinations were not completed by all participants. Alternative neurological, vascular, metabolic, psychiatric, and other causes of dementia were excluded. Each final diagnosis was evaluated by two neurologists and was accepted only when consensus was reached.

HC participants were classified as cognitively normal when they did not meet the education-adjusted abnormality thresholds for objective cognitive impairment, had no cognitive-related loss of independence in daily functioning, and showed no domain-specific neuropsychological evidence of MCI or dementia.

Neuropsychiatric and behavioral symptoms were additionally assessed using the Hamilton Anxiety Rating Scale (HAMA) and Hamilton Depression Rating Scale (HAMD).

### 2.3 Ophthalmic assessment and OCTA imaging

All participants underwent a comprehensive ophthalmic evaluation, including best-corrected visual acuity (BCVA), refractive assessment, intraocular pressure measurement, slit-lamp biomicroscopy, and macular OCTA. The ophthalmic examination and OCTA images were reviewed to exclude ocular conditions that could confound retinal microvascular measurements.

Macular OCTA images were acquired using the Zeiss AngioPlex OCTA system (Carl Zeiss Meditec, Dublin, CA, USA). The device operates at a scanning speed of 68,000 A-scans per second with a central wavelength of 840 nm and incorporates eye-tracking technology to reduce motion artifacts. A 6 × 6-mm scan centered on the fovea was acquired from each eye. Only images with a signal strength index >4 were considered eligible. For each participant, only one eye was included in the analysis; when images from both eyes were eligible, the eye with better overall image quality was selected. Thus, the unit of analysis was the participant rather than the eye. All OCTA examinations were performed by the same experienced examiner.

The analyses focused on the superficial capillary plexus (SCP), defined as the retinal slab extending from the internal limiting membrane (ILM) to the inner plexiform layer (IPL). The macular region was divided into a central 1 × 1-mm subfield, a 3 × 3-mm inner ring, and a 6 × 6-mm outer ring. The inner and outer rings were further divided into superior, inferior, nasal, and temporal quadrants, as illustrated in Supplementary material 1.

Vessel density (VD) was defined as the total vessel length per unit area, whereas perfusion density (PD) was defined as the proportion of the analyzed area occupied by perfused vasculature. The built-in software automatically quantified VD and PD in each macular subfield, together with foveal avascular zone (FAZ) parameters, including FAZ area, perimeter, and circularity.

### 2.4 Statistical analysis

Statistical analyses and model development were performed using R (version 4.3.0) and Python (version 3.11). Continuous variables with a normal distribution are presented as mean ± standard deviation (SD) and were compared between groups using the independent-samples t test. Non-normally distributed continuous variables are presented as median (interquartile range) [M (P25, P75)] and were compared using nonparametric tests. Categorical variables are summarized as counts and percentages and were compared using the chi-square test, Fisher’s exact test, or nonparametric tests, as appropriate. A two-sided *p* value < 0.05 was considered statistically significant.

### 2.5 Model development and validation

#### 2.5.1 Data preprocessing

Prior to model construction, all input features underwent a unified preprocessing pipeline. Missing values were imputed using feature-specific median values, and potential outliers were identified and handled according to prespecified data-quality criteria before model fitting. Given that algorithms such as support vector machines (SVM) and logistic regression are sensitive to feature scale, variables measured on different scales may bias model training and the estimated feature weights. Therefore, z-score standardization was applied at the model input stage, transforming each feature to a distribution with a mean of 0 and a standard deviation of 1.

To prevent information leakage and ensure an unbiased evaluation across Models 1–3, the preprocessing pipeline was fitted within a stratified 5-fold cross-validation framework. Specifically, preprocessing parameters were estimated exclusively from the training set within each fold and then applied to the corresponding test set, thereby maintaining strict independence of the test data from the model-training process.

#### 2.5.2 Stage 1: baseline model using the full OCTA feature set (Model 1)

In the initial stage, a baseline model was constructed to evaluate the overall diagnostic potential of retinal microvascular features. Model 1 used the unified preprocessing pipeline described above before classifier fitting. All 27 extracted quantitative OCTA parameters were included as input features, with a binary outcome label (AD vs. HC). No feature selection or dimensionality reduction was performed at this stage.

Four commonly used machine learning classifiers, logistic regression, SVM, random forest, and extreme gradient boosting (XGBoost), were trained and evaluated on the full feature set. Model performance was estimated using stratified 5-fold cross-validation to preserve the proportion of AD and HC cases in each fold, thereby mitigating the influence of class imbalance on performance assessment. Results from this stage served as the reference baseline for subsequent model optimization.

#### 2.5.3 Stage 2: biomarker selection and optimized model development (Model 2)

To reduce feature redundancy, decrease model complexity, and improve generalizability, an optimized model was developed in Stage 2 by incorporating a feature-selection strategy. Because performing one-time feature selection on the entire dataset may introduce selection bias and information leakage (Varma and Simon, 2006), we implemented a nested cross-validation framework in which feature selection and performance evaluation were conducted independently during model training.

The workflow was as follows:

(1) The entire dataset was split into five stratified folds; in each iteration, four folds were used as the training set and the remaining fold served as an independent test set.
(2) The unified preprocessing pipeline described above was fitted separately within each training fold and applied to the corresponding test fold before feature selection.
(3) Within each training set, recursive feature elimination with cross-validation (RFECV) based on a random forest estimator was used to identify the optimal feature subset. This procedure was conducted exclusively within the training data and remained fully independent of the test set.
(4) Using the selected feature subset, logistic regression, SVM, random forest, and XGBoost classifiers were trained on the training set and evaluated on the corresponding test set.
(5) The set of features selected by RFECV was recorded for each fold. To determine core OCTA biomarkers with stable diagnostic value, we further quantified the selection frequency of each feature across the five nested cross-validation iterations. Features consistently selected in at least four folds (≥80%) were retained as the final core feature panel for subsequent model construction and validation.

#### 2.5.4 Stage 3: multimodal fusion model (Model 3)

In Stage 3, a multimodal fusion model was developed to assess the incremental diagnostic value of demographic and systemic clinical variables beyond OCTA-derived biomarkers. The model integrated the core OCTA features identified in Stage 2 with eight prespecified demographic and clinical variables: age, sex, years of education, smoking status, alcohol consumption, hypertension, diabetes mellitus, and hyperlipidemia. Age and years of education were treated as continuous variables, whereas sex, smoking status, alcohol consumption, hypertension, diabetes mellitus, and hyperlipidemia were encoded as binary indicators. Neuropsychological assessment scores, including MoCA-B, MMSE, and the other cognitive tests, were used for clinical characterization and group classification but were not included as predictors in Model 3.

The combined feature set was subsequently used to train and evaluate four classifiers—logistic regression, SVM, random forest, and XGBoost—under the same stratified five-fold cross-validation scheme used in the previous stages. Model 3 used the same unified preprocessing framework described above before classifier fitting. By comparing the performance of Model 3 with that of Models 1 and 2, we assessed the incremental contribution of demographic and systemic clinical variables to AD classification.

### 2.6 Model performance evaluation

Model diagnostic performance was primarily assessed using the area under the receiver operating characteristic curve (AUC). In addition, sensitivity, specificity, and overall accuracy were calculated, and 95% confidence intervals (CIs) were reported.

To further evaluate the reliability of predicted probabilities and clinical applicability, calibration curves were generated to examine the agreement between predicted probabilities and the observed prevalence. The Brier score was also computed to quantify the accuracy of probabilistic predictions; it ranges from 0 to 1, with lower values indicating better calibration performance.

All performance metrics were derived from the stratified 5-fold cross-validation results to ensure robustness and reproducibility of model evaluation.

The 95% CIs were estimated from the empirical distribution across the stratified cross-validation folds.

### 2.7 Model interpretability analysis

Model interpretability was investigated using Shapley additive explanations (SHAP). Based on game-theoretic principles, SHAP estimates each feature’s marginal contribution to the model output. To examine the contributions of OCTA-derived and clinical variables within the multimodal feature space, SHAP was applied to the Model 3 as an exploratory feature-attribution analysis. SHAP summary (beeswarm) and feature-importance (bar) plots were generated to rank features globally and characterize the direction of their contributions to AD-versus-HC classification.

## 3 Results

### 3.1 Baseline demographic and clinical characteristics

A total of 160 participants were included in the final analysis, comprising 81 HC and 79 patients with AD. Detailed demographic and clinical characteristics are summarized in Table 1. There was no significant difference in age between the two groups (HC: 69.95 ± 7.09 years; AD: 72.01 ± 9.26 years; *p* = 0.115), and sex distribution was also comparable (*p* = 0.634). No significant between-group differences were observed in smoking history, alcohol consumption, or comorbidities including hypertension, diabetes mellitus, and hyperlipidemia (all *p* > 0.05). However, years of education were significantly lower in the AD group than in the HC group (10.05 ± 4.68 vs. 12.03 ± 3.36 years; *p* = 0.003).

**Table 1:** Demographic and baseline clinical characteristics of participants in the HC and AD groups.

Variable	HC (*n* = 81)	AD (*n* = 79)	Statistic	*p* value
Age, years	69.95 ± 7.09	72.01 ± 9.26	*t* = 1.58	0.115
Sex, male/female	39/42(48.1%/51.9%)	42/37(53.2%/46.8%)	*χ*^2^ = 0.23	0.634
Years of education	12.03 ± 3.36	10.05 ± 4.68	***t* = 3.07**	**0.003**
Smoking (yes, %)	21 (25.9%)	24 (30.4%)	*χ*^2^ = 0.16	0.688
Alcohol Consumption	5 (6.2%)	7 (8.9%)	*χ*^2^ = 0.10	0.747
Hypertension (yes, %)	40 (49.4%)	29 (36.7%)	*χ*^2^ = 2.34	0.126
Diabetes (yes, %)	11 (13.6%)	13 (16.5%)	*χ*^2^ = 0.07	0.799
Hyperlipidemia (yes, %)	31 (38.3%)	23 (29.1%)	*χ*^2^ = 1.24	0.265
Intraocular pressure (mmHg)	14.60 ± 1.69	14.38 ± 1.66	*t* = 0.80	0.424
BCVA	0.86 ± 0.19	0.68 ± 0.27	*t* = 4.81	**<0.001**

Continuous variables are presented as mean ± standard deviation (SD) or as median (interquartile range, IQR), as appropriate. Categorical variables are presented as number (percentage). AD, Alzheimer’s disease; HC, healthy control. A two-sided *p* value < 0.05 was considered statistically significant. Bold values indicate *p* < 0.05, denoting statistically significant between-group differences.

### 3.2 Comparison of retinal vessel density between groups

Quantitative OCTA analysis showed that, compared with the HC group, the AD group exhibited significantly reduced VD in the SCP across multiple macular subfields (Table 2).

**Table 2:** Comparison of superficial capillary plexus VD between the HC and AD groups across macular subfields.

Region	HC (*n* = 81)	AD (*n* = 79)	Statistic	*p*
Central (1×1 mm)	4.25 ± 2.65	3.06 ± 2.39	***t* = 2.98**	**0.003**
Inner ring (3×3 mm)	12.65 ± 4.21	10.34 ± 3.91	***t* = 3.60**	**< 0.001**
Outer ring (6×6 mm)	14.01 ± 3.58	12.60 ± 3.20	***t* = 2.63**	**0.009**
Whole macula	13.43 ± 3.61	11.81 ± 3.21	***t* = 3.00**	**0.003**
Nasal inner	12.80 ± 4.61	10.54 ± 3.95	***t* = 3.33**	**0.001**
Nasal outer	16.33 ± 3.90	15.20 ± 3.24	***t* = 2.00**	**0.048**
Temporal inner	12.76 ± 4.47	10.59 ± 4.18	***t* = 3.17**	**0.002**
Temporal outer	11.87 ± 4.01	10.69 ± 3.74	*t* = 1.93	0.056
Superior inner	12.70 ± 4.60	10.53 ± 4.16	***t* = 3.13**	**0.002**
Superior outer	14.32 ± 3.87	12.53 ± 3.66	***t* = 3.01**	**0.003**
Inferior inner	12.37 ± 4.25	9.66 ± 4.46	***t* = 3.93**	**< 0.001**
Inferior outer	13.44 ± 3.97	11.94 ± 3.68	***t* = 2.48**	**0.014**

Data are presented as mean ± SD. VD, superficial capillary plexus vessel density (linear density) in the macular region; AD, Alzheimer’s disease; HC, healthy control. A two-sided p value < 0.05 was considered statistically significant. Bold values indicate *p* < 0.05.

For global metrics, mean VD values in the central subfield, inner ring, outer ring (except for the temporal outer subfield) and the whole macular area were all significantly lower in the AD group than in the HC group (all *p* < 0.01). In the regional analysis, VD was decreased to varying degrees in the AD group in the nasal inner ring, nasal outer ring, temporal inner ring, superior inner ring, superior outer ring, and both inferior inner and inferior outer rings. The most pronounced reductions were observed in the nasal inner ring and inferior inner ring (both *p* < 0.001).

In contrast, VD in the temporal outer ring showed a decreasing trend but did not reach statistical significance (*p* = 0.056). Collectively, these findings suggest widespread retinal microvascular rarefaction in patients with AD, with more prominent alterations in specific macular quadrants/subfields.

### 3.3 Comparison of retinal perfusion density between groups

Consistent with the VD findings, retinal PD also showed a significant decreasing trend in the AD group (Table 3).

**Table 3:** Comparison of superficial capillary plexus PD between the HC and AD groups across macular subfields.

Region	HC (*n* = 81)	AD (*n* = 79)	Statistic	*p*
Central (1 × 1 mm)	0.09 ± 0.06	0.06 ± 0.05	*t* = 3.02	**0.003**
Inner ring (3 × 3 mm)	0.29 ± 0.10	0.24 ± 0.09	*t* = 3.35	**< 0.001**
Outer ring (6 × 6 mm)	0.34 ± 0.09	0.30 ± 0.08	*t* = 2.73	**0.007**
Whole macula	0.32 ± 0.09	0.28 ± 0.08	*t* = 3.02	**0.003**
Nasal inner	0.29 ± 0.11	0.24 ± 0.09	*t* = 3.35	**< 0.001**
Nasal outer	0.39 ± 0.10	0.36 ± 0.09	*t* = 2.10	**0.037**
Temporal inner	0.29 ± 0.11	0.24 ± 0.10	*t* = 3.14	**0.002**
Temporal outer	0.28 ± 0.10	0.25 ± 0.09	*t* = 1.98	0.05
Superior inner	0.30 ± 0.11	0.24 ± 0.10	*t* = 3.14	**0.002**
Superior outer	0.35 ± 0.10	0.30 ± 0.09	*t* = 3.14	**0.002**
Inferior inner	0.29 ± 0.10	0.22 ± 0.11	*t* = 3.35	**< 0.001**
Inferior outer	0.32 ± 0.10	0.28 ± 0.10	*t* = 2.51	**0.013**

Data are presented as mean ± SD. PD, superficial capillary plexus perfusion density in the macular region; AD, Alzheimer’s disease; HC, healthy control. A two-sided *p* value < 0.05 was considered statistically significant. Bold values indicate *p* < 0.05.

Across the central subfield, inner ring, outer ring, and the whole macular area, PD values were significantly lower in the AD group than in the HC group (all *p* < 0.01). Regional analyses further demonstrated markedly reduced PD in the AD group in the nasal inner, nasal outer, temporal inner, superior inner, superior outer, and both inferior inner and inferior outer subfields. The most pronounced differences were observed in the inferior inner and nasal inner subfields (both *p* < 0.001).

PD in the temporal outer subfield was borderline between groups (*p* = 0.05). Overall, these results indicate that patients with AD exhibit not only reduced retinal microvascular structure but also a widespread decline in microcirculatory perfusion.

### 3.4 Comparison of foveal avascular zone parameters between the HC and AD groups

Following the observed reductions in retinal vessel density and perfusion density, we further compared FAZ parameters between the HC and AD groups (Table 4). FAZ area differed significantly between the two groups, with a larger mean area in the AD group than in the HC group (*p* = 0.008). FAZ circularity was also significantly higher in the AD group than in the HC group (*p* < 0.001). Although FAZ perimeter/length was numerically greater in the AD group than in the HC group, the between-group difference did not reach statistical significance (*p* = 0.187). Overall, significant differences between the AD and HC groups were observed for FAZ area and circularity, whereas FAZ perimeter/length remained comparable between the two groups.

**Table 4:** Comparison of foveal avascular zone parameters between the HC and AD groups.

FAZ	HC (*n* = 81)	AD (*n* = 79)	Statistic	*p*
FAZ area	0.26 ± 0.14	0.31 ± 0.15	*t* = 2.71	**0.008**
FAZ perimeter/length	2.13 ± 0.63	2.26 ± 0.54	*t* = 1.33	0.187
FAZ circularity	0.68 ± 0.10	0.72 ± 0.07	*t* = 3.75	**<0.001**

Data are presented as mean ± standard deviation (SD). FAZ, foveal avascular zone; HC, healthy controls; AD, Alzheimer’s disease. *t*, independent-samples *t*-test statistic; *p* values were two-sided. Bold values indicate *p* < 0.05.

### 3.5 Cognitive, neuropsychiatric, and functional characteristics

Compared with the HC group, the AD group exhibited significantly reduced global cognitive performance, as reflected by markedly lower MoCA-B scores (*p* < 0.001) and MMSE scores (*p* < 0.001). In addition, the AD group demonstrated significantly elevated scores on the HAMD, HAMA, Neuropsychiatric Inventory (NPI), and ADL scales relative to the HC group (all *p* < 0.001), indicating greater affective symptoms, neuropsychiatric burden, and functional impairment.

With respect to domain-specific cognitive performance, participants with AD showed significantly poorer outcomes across all assessed cognitive domains. This included memory function (HVLT-0, HVLT-5, HVLT-20, and WMS), language abilities (BNT and VFT), executive function (STT-A and STT-B), and visuospatial skills (ROCFT-copy and ROCFT-recall), with all between-group differences reaching statistical significance (all *p* < 0.001). The detailed results of the neuropsychological assessments are provided in Table 5.

**Table 5:** Neuropsychological and functional characteristics of the HC and AD groups.

Characteristic	HC (*n* = 81)	AD (*n* = 79)	Statistic	*p*
Global cognition				
MoCA-B	24.0 [20.0, 26.0]	10.0 [7.0, 15.0]	*Z* = 9.65	**<0.001**
MMSE	27.0 [26.0, 29.0]	18.0 [13.8, 21.0]	*Z* = 9.56	**<0.001**
Neuropsychiatric symptoms				
HAMD	4.0 [2.0, 6.0]	7.0 [3.5, 10.5]	*Z* = 3.85	**<0.001**
HAMA	4.0 [2.0, 7.0]	7.0 [4.0, 13.5]	*Z* = 3.68	**<0.001**
NPI	0.0 [0.0, 1.0]	1.0 [0.0, 9.0]	*Z* = 4.15	**<0.001**
Activities of daily living				
ADL	15.0 [14.0, 16.0]	22.0 [18.0, 26.0]	*Z* = 8.78	**<0.001**
Memory				
HVLT-0	19.8 ± 5.2	9.2 ± 5.1	*t* = 12.78	**<0.001**
HVLT-5	8.0 [5.0, 9.0]	0.0 [0.0, 0.0]	*Z* = 9.65	**<0.001**
HVLT-20	7.0 [5.0, 9.0]	0.0 [0.0, 0.0]	*Z* = 9.71	**<0.001**
WMS	11.0 [7.5, 12.0]	3.0 [2.0, 5.0]	*Z* = 9.12	**<0.001**
Language				
BNT	24.0 [21.5, 26.0]	17.0 [11.5, 21.0]	*Z* = 7.51	**<0.001**
VFT	13.0 [12.0, 16.5]	8.0 [5.0, 9.0]	*Z* = 8.52	**<0.001**
Executive function				
STT-A	54.0 [43.5, 64.5]	93.0 [66.0, 115.0]	*Z* = 7.91	**<0.001**
STT-B	127.5 [110.5, 149.0]	188.5 [152.8, 212.0]	*Z* = 8.66	**<0.001**
Visuospatial function				
ROCFT-copy	35.0 [33.0, 36.0]	26.0 [13.0, 33.0]	*Z* = 6.05	**<0.001**
ROCFT-recall	17.0 [14.0, 23.5]	0.0 [0.0, 0.0]	*Z* = 9.50	**<0.001**

Data are presented as mean ± SD or median [25th, 75th percentiles], as appropriate. *Z*, standardized test statistic; *t*, *t*-test statistic. HC, healthy controls; AD, Alzheimer’s disease; MoCA-B, Montreal Cognitive Assessment-Basic; MMSE, Mini-Mental State Examination; HAMD, Hamilton Depression Rating Scale; HAMA, Hamilton Anxiety Rating Scale; NPI, Neuropsychiatric Inventory; ADL, activities of daily living; HVLT, Hopkins Verbal Learning Test; HVLT-0, immediate recall; HVLT-5, 5-min delayed recall; HVLT-20, 20-min delayed recall; WMS, Wechsler Memory Scale; BNT, Boston Naming Test; VFT, Verbal Fluency Test; STT, Shape Trail Test; ROCFT, Rey-Osterrieth Complex Figure Test. *p* values were two-sided. Bold values indicate *p* < 0.05.

### 3.6 Model 1: baseline model using the full OCTA feature set

The performance of the baseline models constructed using all OCTA features (Supplementary material 2) is summarized in Figure 1 and Table 6. All four algorithms showed moderate discriminative ability for differentiating AD from HC, with AUC values ranging from 0.772 to 0.829.

**Figure 1:**
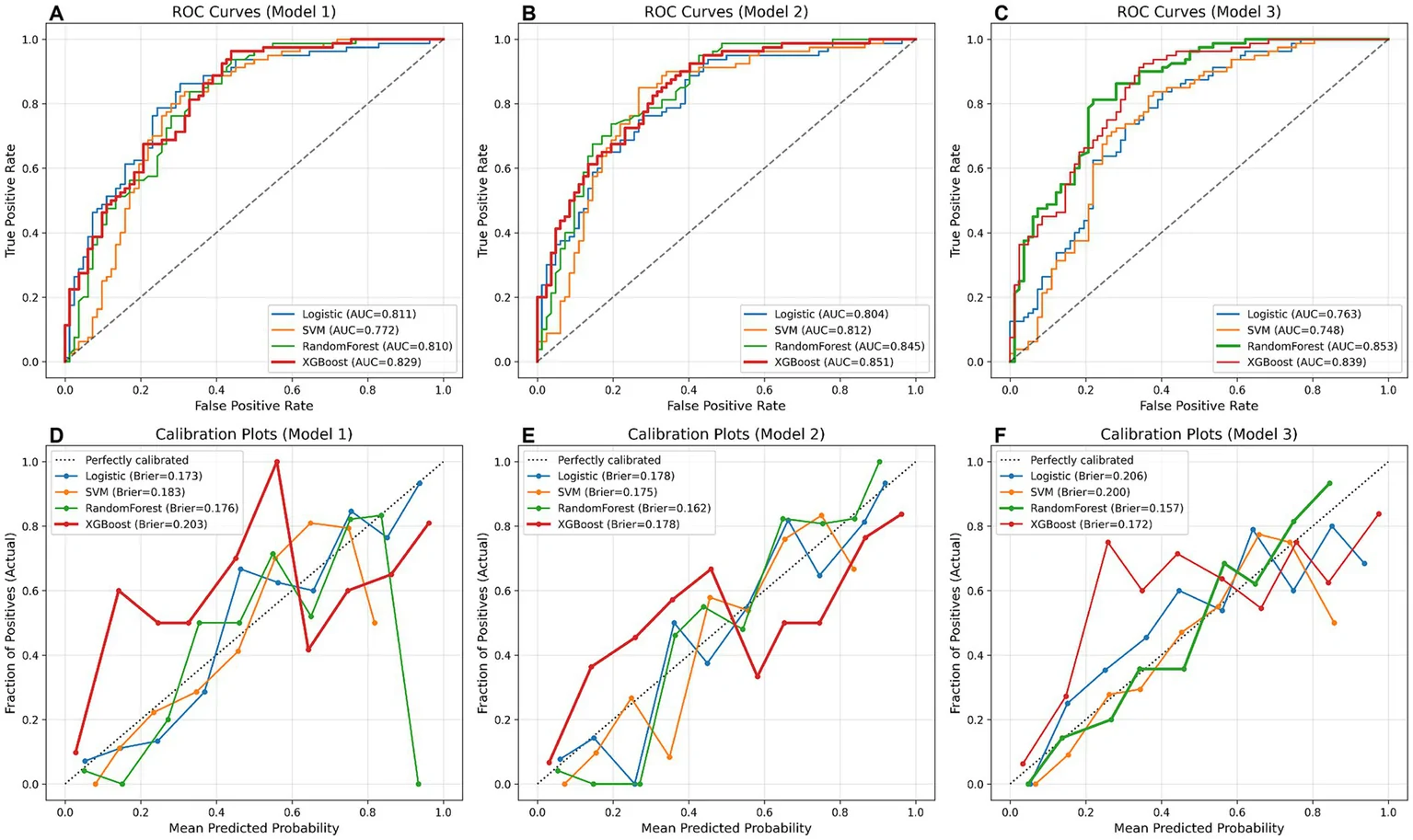
Diagnostic performance and calibration of the three machine-learning model stages. **(A–C)** Receiver operating characteristic curves for Model 1, Model 2, and Model 3 comparing logistic regression, support vector machine (SVM), random forest, and XGBoost classifiers for distinguishing Alzheimer’s disease (AD) from healthy controls (HC). Model 1 used the full OCTA feature set, Model 2 used selected core OCTA features, and Model 3 integrated selected OCTA features with clinical variables. **(D–F)** Calibration plots for the corresponding models; the dotted line indicates perfect calibration, and Brier scores are shown in the legends.

**Table 6:** Performance comparison among different models.

Model	AUC	Sensitivity	Specificity	Accuracy
Model 1				
XGBoost	0.829 (0.751–0.907)	0.738 (0.566–0.859)	0.703 (0.580–0.826)	0.721 (0.616–0.801)
Logistic	0.811 (0.707–0.914)	0.713 (0.609–0.816)	0.763 (0.647–0.878)	0.739 (0.659–0.819)
Random Forest	0.810 (0.716–0.904)	0.775 (0.618–0.932)	0.679 (0.549–0.809)	0.728 (0.627–0.829)
SVM	0.772 (0.690–0.854)	0.813 (0.707–0.918)	0.690 (0.571–0.787)	0.752 (0.668–0.824)
Model 2				
XGBoost	0.851 (0.756–0.946)	0.775 (0.672–0.828)	0.740 (0.546–0.890)	0.758 (0.629–0.839)
Logistic	0.804 (0.674–0.936)	0.737 (0.584–0.866)	0.754 (0.574–0.910)	0.746 (0.614–0.854)
Random Forest	0.845 (0.746–0.924)	0.800 (0.655–0.845)	0.729 (0.508–0.902)	0.765 (0.630–0.826)
SVM	0.812 (0.671–0.923)	0.825 (0.699–0.876)	0.729 (0.571–0.817)	0.777 (0.650–0.831)
Model 3				
XGBoost	0.839 (0.742–0.925)	0.800 (0.652–0.823)	0.728 (0.461–0.923)	0.765 (0.606–0.825)
Logistic	0.763 (0.625–0.906)	0.700 (0.523–0.852)	0.705 (0.530–0.832)	0.703 (0.546–0.824)
Random Forest	0.853 (0.783–0.923)	0.775 (0.749–0.926)	0.729 (0.499–0.960)	0.752 (0.667–0.900)
SVM	0.748 (0.606–0.885)	0.759 (0.605–0.886)	0.753 (0.512–0.812)	0.765 (0.587–0.820)

XGBoost achieved the highest mean AUC (0.829; 95% CI, 0.751–0.907), followed by logistic regression (0.811), random forest (0.810), and SVM (0.772). SVM yielded the highest sensitivity (0.813), whereas logistic regression showed the highest specificity (0.763). Pooled out-of-fold 2 × 2 confusion matrices for all classifiers across Models 1–3 are provided in Supplementary material 3.

### 3.7 Model 2: optimized model based on selected core OCTA biomarkers

#### 3.7.1 Core biomarker selection and model performance

Using RFECV within the nested cross-validation framework, we identified 12 core OCTA features that were selected in at least four of five folds (Figure 2). Performance changes after feature reduction varied across algorithms (Table 6). Compared with Model 1, AUC increased for XGBoost (0.829 to 0.851), random forest (0.810 to 0.845), and SVM (0.772 to 0.812). In contrast, logistic regression showed a slight decrease in AUC from 0.811 to 0.804. Within Model 2, XGBoost achieved the highest AUC (0.851), whereas SVM achieved the highest sensitivity (0.825) and accuracy (0.777). Logistic regression showed mixed changes, with modest increases in sensitivity and accuracy but a slight decrease in specificity. Thus, feature reduction improved several, but not all, performance measures.

**Figure 2:**
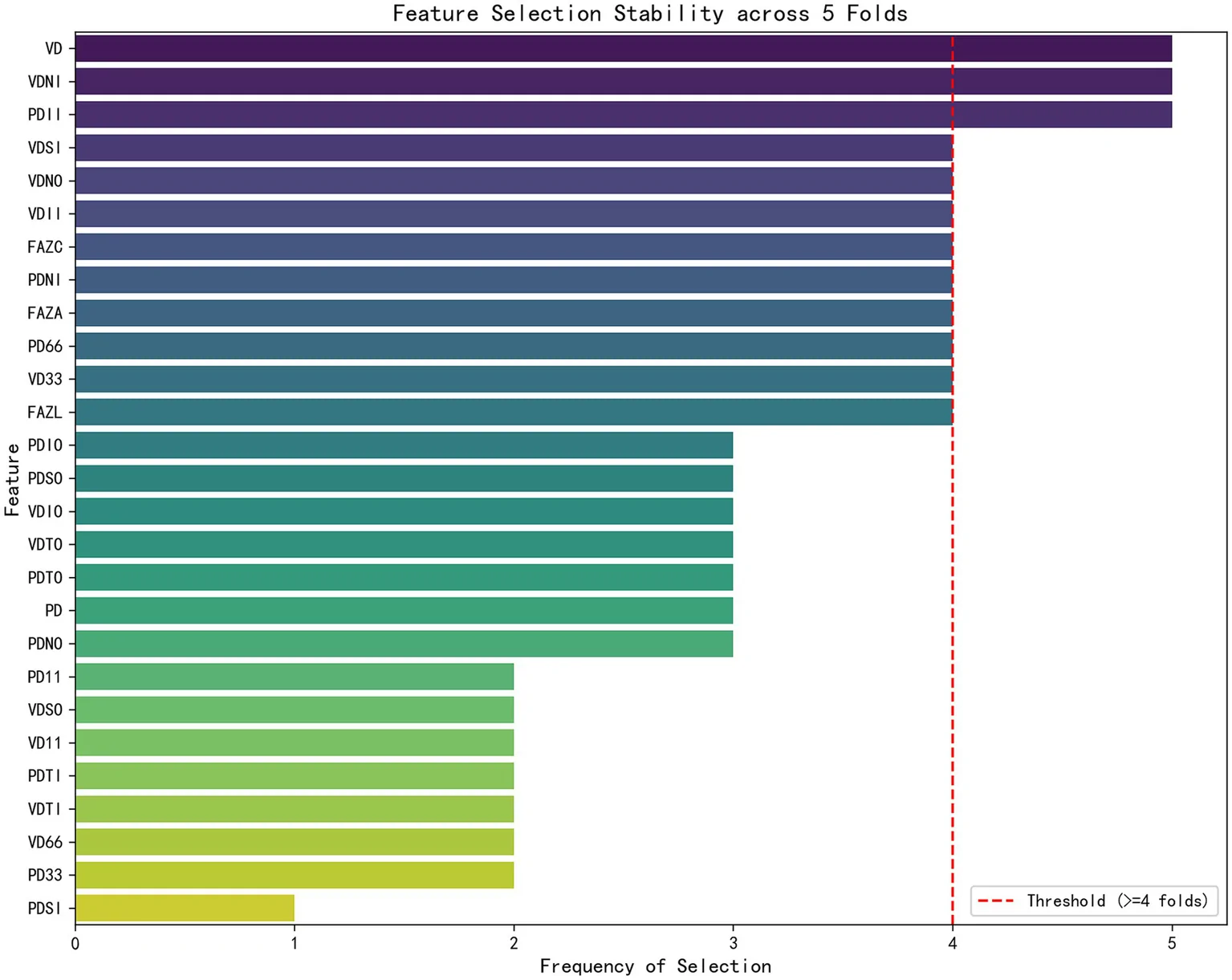
Stability of OCTA feature selection across five cross-validation folds. Horizontal bars show the selection frequency of each candidate feature during recursive feature elimination with cross-validation (RFECV). The red dashed line marks the predefined stability threshold (selection in at least four folds); features meeting this criterion were retained as the core OCTA feature set for Model 2.

### 3.8 Model 3: multimodal fusion model

In Model 3, the 12 core OCTA features were integrated with clinical variables including age, sex,years of education, and medical history(smoking status, alcohol consumption, hypertension, diabetes mellitus, and hyperlipidemia), and the results are summarized in Table 6. Random forest achieved the highest AUC (0.853), whereas XGBoost achieved the highest sensitivity (0.800). SHAP analysis was then performed on the multimodal XGBoost model.

SHAP analysis of the Model 3 XGBoost classifier showed that whole-macula VD had the largest mean absolute SHAP value, followed by vessel density in the nasal inner ring (VDNI), perfusion density in the inferior inner ring (PDII), perfusion density in the 6 × 6 mm outer ring (PD66), foveal avascular zone circularity (FAZC), and vessel density in the nasal outer ring (VDNO). Lower VD and VDNO values were generally associated with positive SHAP values, indicating a shift toward AD predictions. The SHAP distribution for VDNI was heterogeneous, with both positive and negative contributions across observations. Higher PDII, PD66, FAZC, and foveal avascular zone area (FAZA) values generally contributed positively to the AD prediction. Lower years of education were predominantly associated with positive SHAP values, whereas higher years of education tended to contribute negatively. Age and hypertension showed modest positive contributions to the model output. Vessel density in the superior inner ring (VDSI) and sex had relatively small and less consistent effects, whereas smoking and hyperlipidemia contributed only modestly. Diabetes mellitus and alcohol consumption showed near-zero SHAP values. Overall, retinal microvascular features accounted for most of the model importance, while demographic and systemic clinical variables made secondary contributions.

Calibration curves and Brier score analyses showed good agreement between predicted probabilities and observed outcomes across models (Figure 1). SHAP plots further highlighted whole-macula VD as the feature with the largest mean absolute SHAP value (Figure 3).

**Figure 3:**
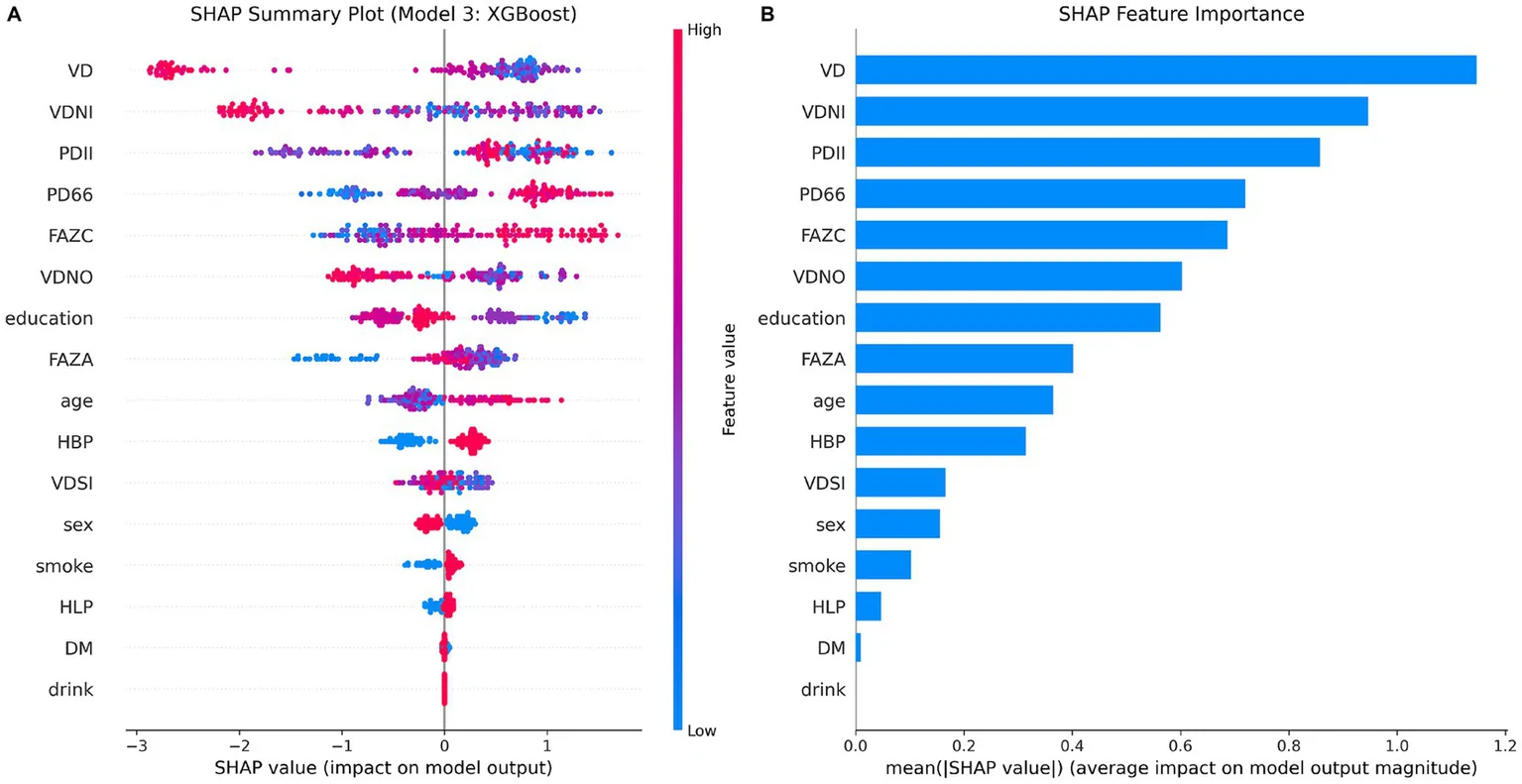
SHAP-based interpretability analysis of the multimodal XGBoost model (Model 3). **(A)** SHAP summary plot showing the direction and magnitude of each feature’s contribution to the model output. Colors indicate feature values ranging from low (blue) to high (red). **(B)** Mean absolute SHAP values showing global feature importance. VD, whole-macula vessel density; VDNI, vessel density in the nasal inner ring; PDII, perfusion density in the inferior inner ring; PD66, perfusion density in the outer ring of the 6 × 6 mm scan; FAZC, foveal avascular zone circularity; VDNO, vessel density in the nasal outer ring; FAZA, foveal avascular zone area; HBP, hypertension; VDSI, vessel density in the superior inner ring; HLP, hyperlipidemia; DM, diabetes mellitus.

## 4 Discussion

This study evaluated OCTA-derived retinal microvascular features with multiple machine-learning models for distinguishing clinically diagnosed AD from HC in a selected cross-sectional case–control cohort. The results showed widespread reductions in VD and PD in the macular SCP, with a regional pattern that was most pronounced in the nasal and inferior quadrants (Bulut et al., 2018; Querques et al., 2019). Furthermore, feature selection reduced the OCTA feature set to 12 stable variables and produced algorithm-dependent changes in diagnostic performance (Guyon et al., 2002).

At the quantitative level, VD and PD in the central region, inner ring, outer ring, and overall macular area were all significantly lower in the AD group than in HC, suggesting that retinal microcirculatory impairment is a pervasive feature of AD. This finding is consistent with previous reports of retinal microvascular degeneration in AD and supports the notion of the retina as a peripheral phenotype of the central nervous system (London et al., 2013). Notably, the microvascular changes were not uniformly distributed; instead, they were more pronounced in the nasal and inferior quadrants. This regional susceptibility may reflect differences in local retinal vascular architecture and metabolic demand.

Regarding model development, the baseline OCTA-only models showed effective discrimination. Reducing the feature set to 12 stable OCTA variables improved AUC for XGBoost, random forest, and SVM, but not for logistic regression. Logistic regression instead showed modest gains in sensitivity and accuracy, together with slight reductions in AUC and specificity. These findings indicate that feature selection reduced model complexity and improved several algorithm-metric combinations, but did not uniformly enhance every classifier. Within Model 2, XGBoost achieved the highest AUC, whereas SVM achieved the highest sensitivity and accuracy. These results underscore the pivotal role of feature engineering in medical imaging-based machine learning (Wisely et al., 2022): removing redundant and unstable features can reduce model complexity and enhance generalizability. The reduced feature set should therefore be viewed as a parsimonious alternative rather than as universally superior to the full-feature model.

Different machine-learning algorithms demonstrated distinct strengths. Among the models based on the core feature set, random forest achieved the best overall discriminative performance, whereas XGBoost showed a relative advantage in sensitivity (Breiman, 2001; Chen and Guestrin, 2016; Steyerberg et al., 2010). The models showed distinct operating characteristics. These results indicate exploratory discrimination between clinically diagnosed AD and HC in the present sample. However, sensitivity and specificity estimated in a case–control design do not establish population-screening performance, particularly when MCI, other dementias, and relevant neurological and ocular differential diagnoses are not represented. The models should therefore be regarded as proof-of-concept classification approaches rather than validated screening or diagnostic tests; their utility for triage or screening requires prospective external validation in clinically representative cohorts.

After adding demographic and clinical variables, the multimodal models did not improve meaningfully over the optimized OCTA model, suggesting limited incremental discrimination from these variables in our cohort. Among the clinical features, years of education remained the most informative and showed a stable inverse association with AD risk, consistent with a cognitive-reserve effect (O'Bryhim et al., 2018; Yeh et al., 2022). Even so, its contribution was smaller than that of the core OCTA microvascular features.

Model interpretability analyses further supported the central role of OCTA-derived microvascular change. Whole-macula VD carried the greatest weight in prediction, and lower values were consistently associated with increased AD risk (Yoon et al., 2019).

Several limitations should be considered when interpreting the findings of this study. First, the cross-sectional design precludes inference about the temporal relationship between retinal microvascular alterations and AD progression. Longitudinal studies are therefore needed to determine whether these features have predictive value. Second, assignment to the AD group was based on clinical criteria without uniform confirmation by CSF biomarkers or PET imaging, which may have introduced misclassification. More importantly, this case–control study compared clinically diagnosed AD with HC and did not include MCI, other dementias, or relevant neurological and ocular differential diagnoses as comparator groups. This restricted participant spectrum may have overestimated classification performance relative to that expected in clinically representative populations. The reported PPV and NPV also reflect the study case proportion and should not be extrapolated directly to populations with different AD prevalence (Steyerberg et al., 2010). Although LR + and LR − are not directly prevalence-dependent, they may still vary according to disease spectrum and participant selection. Third, the between-group difference in BCVA raises the possibility of residual confounding by ocular status or image quality. Participants with significant media opacity or major retinal or ocular disease were excluded, and OCTA scans were required to have a signal-strength index >4. Nevertheless, OCTA-derived VD and PD remain sensitive to signal strength and lens opacity (Yu et al., 2019; Kessler et al., 2023). Because BCVA was not included as a predictor, its independent contribution to model discrimination could not be assessed. Future studies should incorporate BCVA, quantitative image-quality measures, and standardized artifact assessment in covariate-adjusted and sensitivity analyses (Wicklein et al., 2023). Finally, this was a single-center study with a relatively limited sample size. Accordingly, these findings should be regarded as proof-of-concept evidence for AD-versus-HC classification rather than validation of a screening or diagnostic tool. Prospective external validation in consecutive, clinically representative cohorts that include relevant differential diagnoses is required before screening or diagnostic use can be considered.

## 5 Conclusion

In summary, this study demonstrates that OCTA-derived retinal microvascular features may serve as noninvasive markers for AD. By combining XGBoost with an RFECV-based feature-selection strategy, we developed an efficient and parsimonious AD-versus-HC classification model, providing preliminary evidence for further evaluation and supporting the diagnostic value of OCTA-derived retinal microvascular features in AD. However, these findings should not yet be interpreted as validated screening or diagnostic tools. Prospective multicenter studies with representative disease prevalence, relevant differential diagnoses, and independent external validation are needed before clinical use can be assessed.

## Data Availability

The raw data supporting the conclusions of this article will be made available by the authors, without undue reservation.
