# Supplementary material for: A multimodal machine-learning diagnostic model for Alzheimer’s disease: integrating OCTA-derived retinal microvascular features and clinical variables
Source: Front Aging Neurosci. 2026 Sep 23;18:1911592. doi: 10.3389/fnagi.2026.1911592 (PMC13642684; doi:10.3389/fnagi.2026.1911592)
Supplement: Supplementary file 1 [file Data_Sheet_1.DOCX]

**Supplementary Table 1.** Coding reference for OCTA vessel density and perfusion density metrics.

**Coding rule:** VD/PD + **Quadrant** (S/I/N/T, optional) + **Ring** (C/I/O/F)

- **Metric type:** VD = Vessel Density; PD = Perfusion Density
- **Ring:** C = Center (1×1 mm); I = Inner ring (3×3 mm); O = Outer ring (6×6 mm); F = Full/Whole macula (6×6 mm)
- **Quadrant (for inner/outer ring):** S = Superior; I = Inferior; N = Nasal; T = Temporal

| **Feature** | Full name | Definition / Region |
| --- | --- | --- |
| VD11 | Vessel Density – 1×1 mm (Center) | Vessel density in central 1×1 mm |
| VD33 | Vessel Density – 3×3 mm (Inner ring) | Vessel density in inner macular region (3×3 mm) |
| VD66 | Vessel Density – 6×6 mm (Outer ring) | Vessel density in outer macular region (6×6 mm) |
| VD | Vessel Density – Whole macula | Overall vessel density for the full macular scan (device-defined) |
| VDSI | Vessel Density – Superior Inner | Vessel density in superior quadrant of inner ring |
| VDII | Vessel Density – Inferior Inner | Vessel density in inferior quadrant of inner ring |
| VDNI | Vessel Density – Nasal Inner | Vessel density in nasal quadrant of inner ring |
| VDTI | Vessel Density – Temporal Inner | Vessel density in temporal quadrant of inner ring |
| VDSO | Vessel Density – Superior Outer | Vessel density in superior quadrant of outer ring |
| VDIO | Vessel Density – Inferior Outer | Vessel density in inferior quadrant of outer ring |
| VDNO | Vessel Density – Nasal Outer | Vessel density in nasal quadrant of outer ring |
| VDTO | Vessel Density – Temporal Outer | Vessel density in temporal quadrant of outer ring |
| PD11 | Perfusion Density – 1×1 mm (Center) | Perfusion density in central 1×1 mm |
| PD33 | Perfusion Density – 3×3 mm (Inner ring) | Perfusion density in inner macular region (3×3 mm) |
| PD66 | Perfusion Density – 6×6 mm (Outer ring) | Perfusion density in outer macular region (6×6 mm) |
| PD | Perfusion Density – Whole macula | Overall perfusion density for the full macular scan (device-defined) |
| PDSI | Perfusion Density – Superior Inner | Perfusion density in superior quadrant of inner ring |
| PDII | Perfusion Density – Inferior Inner | Perfusion density in inferior quadrant of inner ring |
| PDNI | Perfusion Density – Nasal Inner | Perfusion density in nasal quadrant of inner ring |
| PDTI | Perfusion Density – Temporal Inner | Perfusion density in temporal quadrant of inner ring |
| PDSO | Perfusion Density – Superior Outer | Perfusion density in superior quadrant of outer ring |
| PDIO | Perfusion Density – Inferior Outer | Perfusion density in inferior quadrant of outer ring |
| PDNO | Perfusion Density – Nasal Outer | Perfusion density in nasal quadrant of outer ring |
| PDTO | Perfusion Density – Temporal Outer | Perfusion density in temporal quadrant of outer ring |

**Supplementary Table 2.** Coding reference for foveal avascular zone (FAZ) metrics.

| Feature | full name | Definition / notes |
| --- | --- | --- |
| FAZA | FAZ Area | Area of the foveal avascular zone (typically mm²) |
| FAZL | FAZ Length | FAZ boundary length/perimeter as exported by the device (typically mm) |
| FAZC | FAZ Circularity | Circularity index of FAZ (unitless; typically 0–1, higher = more circular) |
