# Supplementary material for: A multimodal machine-learning diagnostic model for Alzheimer’s disease: integrating OCTA-derived retinal microvascular features and clinical variables
Source: Front Aging Neurosci. 2026 Sep 23;18:1911592. doi: 10.3389/fnagi.2026.1911592 (PMC13642684; doi:10.3389/fnagi.2026.1911592)
Supplement: Supplementary file 2 [file Supplementary_file_1.DOCX]

**Summary of confusion matrices for various models and classifiers**

The study employs a stratified five-fold cross-validation to evaluate the model, with each fold yielding a set of test set prediction results. To present a fair 2x2 table reflecting overall performance, we summarize the prediction results of all test set samples from the five-fold cross-validation

| **Model** | **classifier** | **TP** | **FP** | **FN** | **TN** | **N** |
| --- | --- | --- | --- | --- | --- | --- |
| Model 1 | XGBoost | 58 | 24 | 21 | 57 | 160 |
|  | Logistic | 56 | 19 | 23 | 62 | 160 |
|  | Random Forest | 61 | 26 | 18 | 55 | 160 |
|  | SVM | 64 | 25 | 15 | 56 | 160 |
| Model 2 | XGBoost | 61 | 21 | 18 | 60 | 160 |
|  | Logistic | 58 | 20 | 21 | 61 | 160 |
|  | Random Forest | 63 | 22 | 16 | 59 | 160 |
|  | SVM | 65 | 22 | 14 | 59 | 160 |
| Model 3 | XGBoost | 66 | 21 | 13 | 60 | 160 |
|  | Logistic | 55 | 24 | 24 | 57 | 160 |
|  | Random Forest | 61 | 22 | 18 | 59 | 160 |
|  | SVM | 60 | 20 | 19 | 61 | 160 |

Note:

TP = True positive (AD) and predicted positive (AD);

FP = False positive: participants who were actually HC but predicted as AD;

FN = True AD but predicted as HC;

TN = True HC and predicted as HC.

**Summary of predictive values and likelihood ratios for various models and classifiers**

PPV, NPV, pLR, and nLR were calculated for each classifier in Models 1-3.

| Model | classifier | PPV | NPV | pLR | nLR |
| --- | --- | --- | --- | --- | --- |
| Model 1 | XGBoost | 0.707 | 0.731 | 2.478 | 0.378 |
|  | Logistic | 0.747 | 0.729 | 3.022 | 0.380 |
|  | Random Forest | 0.701 | 0.753 | 2.406 | 0.336 |
|  | SVM | 0.719 | 0.789 | 2.625 | 0.275 |
| Model 2 | XGBoost | 0.744 | 0.769 | 2.978 | 0.308 |
|  | Logistic | 0.744 | 0.744 | 2.973 | 0.353 |
|  | Random Forest | 0.741 | 0.787 | 2.936 | 0.278 |
|  | SVM | 0.747 | 0.808 | 3.029 | 0.243 |
| Model 3 | XGBoost | 0.759 | 0.822 | 3.222 | 0.222 |
|  | Logistic | 0.696 | 0.704 | 2.350 | 0.432 |
|  | Random Forest | 0.735 | 0.766 | 2.843 | 0.313 |
|  | SVM | 0.750 | 0.762 | 3.075 | 0.319 |

Note:

PPV, positive predictive value;

NPV, negative predictive value;

pLR, positive likelihood ratio;

nLR, negative likelihood ratio.
