# Supplementary material for: A multimodal machine-learning diagnostic model for Alzheimer’s disease: integrating OCTA-derived retinal microvascular features and clinical variables
Source: Front Aging Neurosci. 2026 Sep 23;18:1911592. doi: 10.3389/fnagi.2026.1911592 (PMC13642684; doi:10.3389/fnagi.2026.1911592)
Supplement: Supplementary file 3 [file Table_1.DOCX]

**Supplementary Figure 1.** Macular 9-sector grid based on the ETDRS layout, showing the central foveal subfield (1 x 1 mm), inner ring (3 x 3 mm), outer ring (6 x 6 mm), and quadrant definitions (superior, inferior, nasal, and temporal).
